# Molecular and serological prevalence of *Leptospira* spp. among slaughtered cattle and associated risk factors in the Bahr El Ghazal region of South Sudan

**DOI:** 10.1186/s12917-024-04154-0

**Published:** 2024-07-06

**Authors:** David Onafruo, Jörn Klein, Joseph Erume, Clovice Kankya, Ambrose Jubara, Ikwap Kokas, Terence Odoch, Musso Munyeme, Lordrick Alinaitwe, Estella Kitale, Peter Marin, Esther Sabbath, Anou Dreyfus

**Affiliations:** 1 Department of Biosecurity, Ecosystem and Veterinary Public Health (BEP), College of Veterinary Medicine Animal Resources and Biosecurity (COVAB), Makerere University, P.O Box 7062, Kampala, Uganda; 2 Department of Nursing and Health Sciences, Faculty of Health and Social Sciences, University of South -Eastern (USN), and Department of Microsystems, Faculty of Technology, Natural Sciences and Maritime Sciences, University of South-Eastern (USN), PO BOX 235, Porsgrunn, Norway; 3 Department of Biotechnical and Diagnostic Sciences, Faculty of Veterinary Medicine Animal Resources and Biosecurity (COVAB), Makerere University, P.O Box 7062, Kampala, Uganda; 4 Department of Clinical Studies, Faculty of Veterinary Science, University of Bahr El Ghazal (UBG), P.O Box 30, Wau, South Sudan; 5 Department of Biomolecular Resources and Biolab Sciences, College of Veterinary Medicine Animal Resources and Biosecurity (COVAB), Makerere University, P.O Box 7062, Kampala, Uganda; 6 Africa Centre of Excellent for Infectious Diseases of Humans and Animals, School of Veterinary Medicine, University of Zambia, P.O Box 32379, Lusaka, Zambia; 7 Central Diagnostic Laboratory, College of Veterinary Medicine Animal Resources and Biosecurity (COVAB), Makerere University, P.O Box 7062, Kampala, Uganda; 8 Department of Public Health, College of Public and Environmental Health, University of Bahr El Ghazal (UBG), P.O Box 30, Wau, South Sudan; 9 Section of Epidemiology, Vetsuisse Faculty, University of Zurich, P.O Box 1931, Zurich, Switzerland

**Keywords:** *Leptospira*, Serology, Real-time PCR, South Sudan

## Abstract

**Introduction:**

Leptospirosis is a neglected emerging and zoonotic disease reported worldwide. This study sought to determine the molecular and serological prevalence of *Leptospira* spp. and the associated risk factors in slaughtered cattle from the Bahr El Ghazal region of South Sudan.

**Materials and methods:**

Between January 16th and February 25th, 2023, blood and urine samples were collected from 402 cattle at the Lokoloko Municipal Slaughterhouse in Western Bahr El-Ghazal State. Serum samples were tested using the microscopic agglutination test (MAT), with a panel of 12 serovars (sv) from 12 serogroups (sg) and 4 species (spp) of *Leptospira* spp. These serovars had been previously identified in Sudan and the East African region. Simultaneously, 400 corresponding urine samples were screened using qualitative real-time polymerase chain reaction (PCR) to detect the shedding of *Leptospira* spp. in urine. To identify the associated risk factors, the age, sex, breed and body condition score of each sampled cattle was noted at the time of sampling and subsequently analysed using logistic regression models.

**Results:**

Among the 402 serum samples screened, a substantial 81.8% (329/402, 95% CI 77.9–85.3) displayed seropositivity for *Leptospira* spp. with a MAT titre ≥ 100. The prevalence of urine shedding determined by PCR was 6% (23/400, 95% CI 3.8–8.4), while probable recent leptospirosis with a MAT ≥ 1:800 was observed in 33.1% (133/402, 95% CI 28.6–37.8) of the cattle. Multiple reactions were detected in 34.8% (140/402, 95% CI 30.6–39.5) serum samples. The seropositivity was against *L. borgpetersenii* sg. Tarassovi (78.6%; 316/402, 95% CI 74.4–82.3), followed by *L. borgpetersenii* sg. Ballum at 20.4% (82/402, 95% CI, 16.7–24.4%), *L. kirschneri* sg. Autumnalis At 8.7% (35/402, 95% CI 5.7–11.7), *L. interrogans* sg. of Pomona at 7.0% (28/402, 95% CI 4.5–9.5), and *L. interrogans* sg. Hebdomadis was 5.0% (20/402, 95% CI 2.8–7.2). Several risk factors are associated with seropositivity. Older animals (≥ 2 years) had 2.0 times greater odds (95% CI 1.14–3.5) of being seropositive than younger animals (< 2 years), P-value = 0.016. Female animals demonstrated 2.1 times greater odds (95% CI 1.2–3.6) of seropositivity than males did (P-value = 0.008). Additionally, Felata/Mbororo cattle exhibited 2.4 times greater odds (95% CI 1.3–4.5) of being seropositive than did local Nilotic cattle (P-value = 0.005). The agreement between the MAT and PCR results was poor, as indicated by a kappa statistic value of 0.001 and a P-value of 0.913. But there was a moderate agreement between MAT high titres ≥ 800 and PCR positivity with a kappa statistic value = 0.501 and a P-value < 0.001.

**Conclusion:**

In addition to the high seroprevalence, *Leptospira* spp. were found in the urine of slaughtered cattle, suggesting that leptospirosis is endemic to the study area. This finding underscores the significance of cattle as potential sources of infection for slaughterhouse workers, the general public, and other animal species. To address this issue effectively in the Bahr El Ghazal Region and South Sudan, a comprehensive strategy involving a multidisciplinary approach is essential to minimize disease among animals, hence reducing potential zoonotic risks to humans.

## Introduction

Leptospirosis is a neglected emerging and zoonotic disease reported worldwide. This disease has productive and reproductive impacts on infected livestock and significant health risks for humans when they are exposed to infected animals or contaminated environment, highlighting the importance of leptospirosis in the context of One Health [1–3]. Transmission in cattle occurs primarily indirectly from the contaminated environment (soil and water) with urine or direct contact with infected animals [2, 4, 5].

Leptospirosis poses a direct threat to human health and results in economic losses in livestock. Cattle are considered socioeconomic assets and potential hosts for various pathogenic *Leptospira* spp. serovars that cause infection in abattoir workers and livestock farmers [2, 6, 7]. Leptospirosis in cattle is characterized by production and reproduction disorders, in form of abortion storms, stillbirths, reduced milk production, with a possible lethal outcome in acute forms, especially in calves and foetuses characterised by meningitis [2, 8]. Clinical diagnosis of the acute form of the disease is difficult. It may be misleading due to the nonspecific signs and symptoms that are also shared with other infections, such as babesiosis and anaplasmosis, both of which are prevalent in many tropical and subtropical countries where leptospirosis is endemic [9]. Leptospires can be detected in the urine of serologically negative cattle, necessitating the use of other associated techniques, such as polymerase chain reaction (PCR), to increase the efficiency of diagnosis [10, 11].

The microscopic agglutination test (MAT) is the serological gold standard test for the diagnosis of leptospirosis, but it has several limitations, such as the inability to detect infection in the early phase of the disease, due to the absence of detectable antibodies in the first week after onset of symptoms. Further, live bacteria are used in the diagnostic panel for antigens, and well-trained experts are needed to conduct tedious laboratory work. To detect an acute infection paired samples tested at a two*-*week intervals are required [12, 13]. PCR is a robust alternative to MAT for the diagnosis of leptospirosis in the acute phase of the disease [12].

A study conducted in 1989 in the Melut Upper Nile District of South Sudan revealed a 63.5% seroprevalence of *Leptospira* spp. in cattle using the reference microscopic agglutination test (MAT), which used 13 serovars from 13 serogroups [14]. For the Bahr El Ghazal region, a major cattle-keeping region of South Sudan that hosts 12 million heads of cattle, representing more than 50% of the national livestock population [15], we could not find any published data on leptospirosis in cattle using either serological or molecular methods. Therefore, this research sought to assess the presence of leptospires in slaughtered cattle by detecting their DNA in urine through PCR as an indirect diagnostic test to establish presence of leptospires shedding in the urine of cattle, and to identify exposure via a serological method (MAT). A further objective was the analysis of the agreement between MAT and PCR. Additionally, this study aimed to explore the associated risk factors for the *Leptospira* spp. seroprevalence in cattle in the Bahr El Ghazal region.

## Materials and methods

### Study site, design, and population

**Fig. 1:**
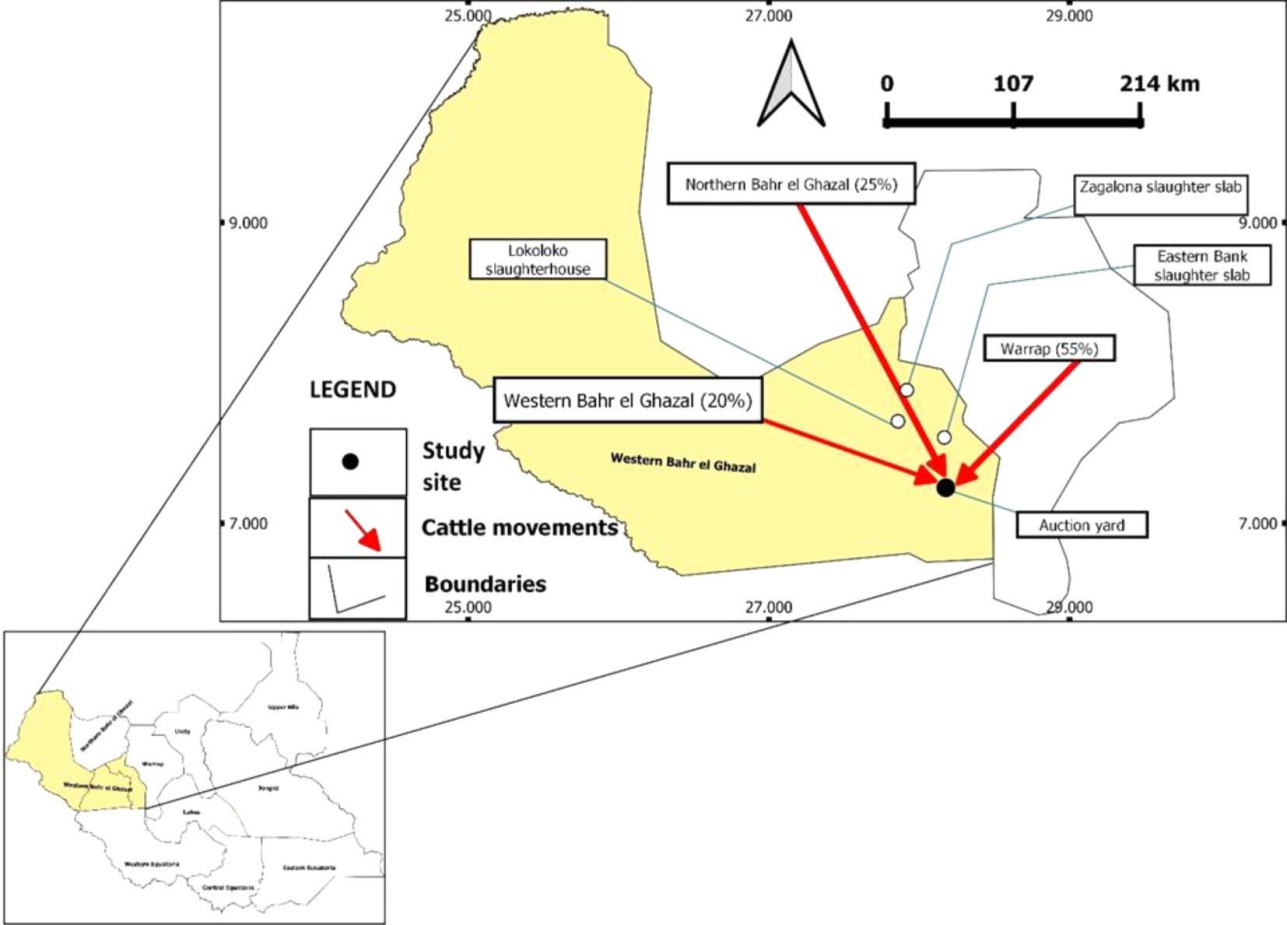
Map of South Sudan, highlighting the study area (in light yellow) and cattle movement within the region (in red). The auction yard is represented by a black circle. The study investigated the molecular and serological prevalence of *Leptospira* spp. among slaughtered cattle (*n* = 402) at the Lokoloko slaughterhouse, marked by a white circle, from January to February 2023

This cross–sectional study was carried out in the Wau Municipal Council, Western Bahr El Ghazal State, Bahr El Ghazal Region of South Sudan, from 16 January to 25 February 2023. Sundays were skipped. The study population was cattle brought by traders for slaughter at the Lokoloko municipal slaughterhouse in the Western Bahr El-Ghazal State. The traders buy cattle from the main livestock auction kraal on the Eastern Bank, where livestock from all over the geographical region of Bahr El Ghazal (Western Bahr El Ghazal, Northern Bahr El Ghazal, and Warrap States) are collected and brought to the auction kraal in the Western Bahr Ghazal State (Fig. 1). The Lokoloko slaughterhouse was purposively selected based on its number of slaughtered cattle, which is the highest within the whole region, ranging from 47 to 50 cattle per day (approximately 1500 cattle per month).

Slaughtered cattle were restrained and laid down on different slaughtering lines, which enabled us to easily select them during the slaughtering process and collect blood and urine samples. Since the Principal Investigator and the research assistants sampled individual animals brought to the slaughterhouse, which originated from different farms by individual traders from within the Bahr El Ghazal region, including Mbororo cattle breed from West African countries but not herds, clustering was not considered. Animals brought from different farms and herds are first mixed at the auction, and after they are bought as individual animals by butchers, they are also mixed for the second time at the slaughterhouse. The data on the origin of the animals were sensitive because of the widespread cattle raiding within the region and South Sudan, making it difficult to trace the spatial distribution of *Leptospira* spp. prevalence in cattle.

### Sample size estimation and sampling strategy

The sample size for estimating the apparent prevalence of *Leptospira* spp. seroprevalence was based on a tested prevalence of 63.5% [14] determined via systematic random sampling of the finite population of 1,500 individuals; an imperfect test was used at the 95% confidence level and a precision of 0.05; and 359 cattle blood and urine samples were estimated using Ausvet Epitools calculators [16].

To increase the power level of the study and to cover for nonresponse and missing values as recommended by [17], the sample size was oversampled to 402. The 400 urine samples were sufficient to investigate disease activity in cattle via PCR based on the estimated 5.8% shedding prevalence among slaughtered cattle from Uganda reported by Alinaitwe et al. [18]. Systematic random sampling was applied to select the slaughtered cattle until the desired sample size of 402 was reached based on the sampling strategy used by Alinaitwe et al. [18]. In brief, our slaughterhouse had four slaughtering lines. Samples were systematically collected from those four lines each day. Out of 50 cattle slaughtered per day, a maximum of 13 cattle were selected and sampled per day for one month.

The first animal was randomly picked from each slaughtering line, and thereafter, every fourth animal (adding 4 to the previously picked number) was chosen until the sample size was achieved.

### Sample and data collection

Approximately 10 mL of blood was aseptically collected from each animal by free capture into gel-activated yellow-capped tubes at the point of the slit to bleed the animal. After the carcass was flayed and opened, corresponding urine samples of approximately 20 mL were collected directly from the same animal’s urinary bladder using a sterile syringe and needle into a sterile 60 mL urine container (Bioset, Medic plastic). Each animal was given a unique ID, which was on the labelled tube. The following data were recorded for each animal: breed (local = Nilotic vs. foreign = Felata/Mbororo), sex (male vs. female), age (adult vs. young), and body condition score (BCS) (poor vs. good vs. very good). The breed of cattle was classified into two local Nilotic breeds and one local Mbororo/Felata breed from West Africa, which were kept by nomadic pastoralists [19, 20]. The sex of the animals, both male and female, was also captured from the cattle brought to the slaughterhouse. Age was estimated employing tooth eruption [21], two age groups were considered: less than 2 years old and greater than or equal to 2 years old. (BCS) was classified into three categories by visual examination of fat cover palpation over the cattle’s body as poor, good, or very good following the description given by [22].

All blood and urine samples were kept in a cool box on ice for each day’s field collection and transported to the laboratory at the University of Bahr El Ghazal (UBG) for processing. Blood samples were centrifuged at 5000 rpm at room temperature for 5 min, and sera were aliquoted into 2 mL cryovials. 12 mL of urine sample was aliquoted into a falcon tube, centrifuged at 15,600xg for 15 min at room temperature (24^°^C), supernatant fluids were decanted, 12 mL phosphate-buffered saline (PBS), was added to the sedimentation, centrifuged at 15,600xg for 15 min supernatant fluids decanted, then 600 µL PBS was added and the mixture aliquoted into cryovial.

All the samples were stored in a -20 °C freezer in the Wau Teaching Hospital Expanded Program for Immunization (EPI) laboratory. Urine and serum samples were transported by air from South Sudan to Uganda and stored at -80 °C before laboratory analysis at the Central Diagnostic Laboratory (CDL) College of Veterinary Animal Resources and Biosecurity (COVAB), Makerere University, Uganda.

### Laboratory diagnosis

#### Serological testing

The microscopic agglutination test (MAT) was used to detect antibodies against *Leptospira* spp. since it is the gold standard test for distinguishing *Leptospira* spp. serogroups in cattle, as described by OIE standards [23]. A panel of 12 serovars (sv) from 12 different serogroups (sg), four *Leptospira* species (Table 1), and the corresponding panel of polyclonal rabbit reference antisera representing twelve serogroups were imported from Amsterdam, University Medical Centre “UMC”, Academic Medical Centre (Leptospirosis Reference Centre), Netherlands. The selection of the panel was performed according to the predominant serovars reported by Sebek et al. (1989) from Sudan and within East African countries, e.g., Uganda [24–27], Kenya [28], Tanzania [29], and Ethiopia [30, 31]. serovar cultures from seven-day-old live *Leptospira* spp. were used to screen the serum samples at an initial dilution of 1:50. All positive reactions including reactions to more than one serovar and serogroup were noted, the positive reacting samples were then further titrated via 2-fold dilution to determine the endpoint titre, defined as the highest serum dilution capable of agglutinating ≥ 50% of leptospires [32]. A MAT positive sample was one that reacted to at least one serogroup at a titre ≥ 100. In instances where a sample reacted to multiple serogroups, the titres of all were reported.

Sera with a titre ≥ 100 against any *Leptospira* serogroup were considered seropositive [13]. An antiserum standard (positive control) for each of the serogroups tested was included. It is important to compare the agglutination in the positive sample with the negative control (PBS). The positive controls were used to help compare the agglutination patterns in the positive sample.

**Table 1:** Panel of *Leptospira* spp., used as live antigens in the microscopic agglutination test

Genomospecies	Serogroups	Serovars	Strains
*L. interrogans*	Icterohaemorrhagiae	Icterohaemorrhagiae	RGA
	Pomona	Pomona	Pomona
	Hebdomadis	Hebdomadis	Hebdomadis
	Australis	Australis	Ballico
	Canicola	Canicola	Strain Hond Utrecht IV
*L. borgpetersenii*	Sejroe	Sejroe	M84
	Ballum	Kenya	Njenga
	Pyrogenes	Nigeria	Vom
	Tarassovi	Tarassovi	Perepelitsin
*L. kirschneri*	Autumnalis	Butembo	Butembo
	Grippotyphosa	Grippotyphosa	Duyster
*L. weilli*	Celledoni	Celledoni	Celledoni

### Molecular diagnosis

#### DNA extraction from urine

The thawed urine pellets (200 µL) were mixed with PBS to reach a volume of 2 mL in an Eppendorf tube, then centrifuged at 4°C for 15 minutes. The supernatant was discarded, and the remaining pellet was resuspended in 200 µL of PBS for DNA extraction using a Qiagen DNeasy Blood and Tissue Kit following the manufacturer’s guidelines. The extracted DNA was eluted and stored at -20°C”.

#### Real-time polymerase chain reaction (PCR)

A TaqMan real*-*time PCR (PCR) was used to detect specific pathogenic *Leptospira* species. PCR was used to amplify the *lipL32* gene with primers and probes as previously reported [33]. The PCR conditions were validated using a dilution series of *L. interrogans* serovar Icterohaemorrhagiae strain RGA, which yielded an efficiency of 100% and 101.6% on the 7500 Fast according to the Step One Plus^®^ PCR System (Applied Biosystems). The detection limit was 10 genome equivalents per reaction, the ideal threshold was 0.06, and the cut-off was 45 cycles. No false-positive reactions were observed during the validation process. All the reactions were carried out in duplicate on *a* Step One Plus^®^ with the recommended default cycling settings (holding at 50 °C for 2 min, 95 °C for 10 min, and 40 cycles of 95 °C for 15 s and 60 °C for 1 min). The final concentrations of the mixture (20 µL) were as follows: 1×TaqMan^®^ Universal PCR Master Mix, No AmpErase UNG^®^, 0.5×TaqMan^®^ Exogenous Internal Positive Control mix (IPC), 0.5× IPC template (Applied Biosystems), 1 µM of each primer, 80 nM of the probe, and 2.0 µL of template. For each run, DNA from the *L. interrogans* serovar Icterohaemorrhagiae strain RGA was included as a positive control, and pyrogen‐free water was used as a negative control. The IPC made it possible to control for inhibition and thus prevent false‐negative results. Samples were considered positive when they showed an exponential amplification curve at cycle times < 40, with the threshold set at 0.06 as previously described [18, 34].

### Data analysis

Cattle demographic data and molecular and serological test results were coded and entered into Microsoft Excel 2016 (Microsoft Corp., Redmond, WA, USA). The data were analysed using SPSS software (IBM SPSS statistics version 26) and epitool calculators. A *Leptospira* spp. seropositive case was defined as a MAT titre ≥ 100 against any serovar [35]. A fourfold increase in the MAT titre ≥ 800 confirmed acute probable recent leptospirosis in the animals [36, 37]. Urine samples were considered positive if the PCR ct value was < 40. Our outcome variable of interest was the overall molecular or serological prevalence of *Leptospira* spp. in the urine or serum of the slaughtered cattle. The associations between overall seroprevalence and the different exposure variables (age, sex, breed, and BCS of the slaughtered cattle) were analysed using bivariate logistic regression.

Furthermore, multivariable logistic regression analysis was performed by selecting the manual backward Wald method to assess the association between these exposure variables and *Leptospira* spp. seropositivity while controlling for the effect of other variables. Exposure variables were each entered into the model if the bivariable P - value was ≤ 0.2 and were kept in the model if the likelihood ratio test was statistically significant (P -value ≤ 0.05). The agreement between MAT and PCR positive results was assessed using Cohen’s Kappa statistic and was interpreted as follows; < 0.2: slight agreement, 0.2–0.4: fair agreement, 0.4–0.6: moderate agreement, 0.6–0.8: substantial agreement, 0.8–0.9: perfect agreement [38]. And with Fleiss-adjusted (95% CIs) [39].

Additionally, we analysed the correlation between samples with high MAT titres ≥ 800 (probable recent leptospirosis) and PCR positive results.

## Results

### Demographic characteristics and Leptospira spp. seroprevalence among slaughtered cattle

Of the 402 screened cattle, 81.8% (329/402, 95% CI 77.9–85.3) were found to have antibodies against at least one of the twelve serovars/serogroups detected at a MAT titre ≥ 100. Among the sampled slaughtered cattle, the majority (68.4%, 275/402) were adult animals (≥ 2 years), and 84.4% were seropositive (232/402, 95% CI 79.9–89.1). Conversely, the population of younger animals (< 2 years) accounted for 31.6% (127/402) of the population, and 76.4% of the population was seropositive (97/127, 95% CI 68.9–84.2) against *Leptospira* spp. According to the sex distribution, 45.8% (218/402) were female cattle, for which 87.5% were seropositive (161/184, 95% CI 82.4–92.0). Moreover, the study population comprised 64.7% (260/402) of the Nilotic local breed and had 78.8% seropositivity (205/260, 95% CI 74.1–84.0). In comparison, the Felata (Mbororo) breed accounted for 35.3% (142/402) of the population and had an 87.3% seropositivity (124/142, 95% CI 80.8% − 92.4). Additionally, 20.6% (83/402) of the slaughtered cattle were identified as having poor body condition and exhibited 88.0% seropositivity (73/83, 95% CI 80.7–94.5) against *Leptospira* spp. (Table 2).

**Table 2:** Demographic characteristics of the sampled cattle (*N* = 402), *Leptospira spp*., percentage of seropositive individuals, and 95% confidence intervals (CIs) for seropositivity against any sg

Variables	Categories	*n* (%)	Number positive (Prevalence%)	95% C.I.	
				Lower	Upper
Age	Young (< 2 years)	127(31.6)	97(76.4)	68.9	84.2
	Adult (≥ 2 years)	275(68.4)	232(84.4)	79.9	89.1
Sex	Male	218(54.2)	168(77.1)	70.8	83.0
	Female	184(45.8)	161(87.5)	82.4	92.0
Breed	Nilotic	260(64.7)	205(78.8)	74.1	84.0
	Felata/Mbororo	142(35.3)	124(87.3)	80.8	92.4
BCS	Poor	83(20.6)	73(88.0)	80.7	94.5
	Good	296(73.6)	236(79.7)	75.2	84.3
	Very good	23(5.7)	20(87.0)	71.4	100
Overall *Leptospira* spp. seroprevalence		402(100)	329(81.8)	77.9	85.3

BCS = body condition score, CI = confidence interval, N = study population size

### Seroprevalence of specific Leptospira spp. serovars

The predominant seroreactivity against sv. Tarassovi sg. Tarassovi was detected at 78.6% (316/402, 95% CI 74.4–82.3) of the study population. This was followed by sv. Kenya sg. Ballum at 20.4% (82/402, 95% CI 16.7–24.4), sv. Butembo sg. of Autumnalis at 8.7% (35/402, 95% CI 5.7–11.7), vs. Pomona sg. Pomona at 7.0% (28/402, 95% CI 4.5–9.5), and sv.Hebdomadis sg Hebdomadis had 5.0% (20/402, 95% CI 2.8–7.2). The prevalence of other serovars, such as sv Sejroe sg. Sejroe at 2.2% (9/402, 95% CI 1.0–4.0), sv. Grippotyphosa sg. Grippotyphosa at 2.0% (8/402, 95% CI 0.7–3.2), sv Nigeria sg. Pyrogenes at 1.7% (7/402, 95% CI 0.7–3.0), sv. Australis sg. of the Australian population at 1.0% (4/402, 95% CI 0.0–2.2), and sv. Canicola sg. Canicola at 0.2% (1/402, 95% CI, 0.0*–*1.0). No animals tested positive for sv. Icterohaemorrhagiae sg. Icterohaemorrhagiae and sv. Celledoni sg. Celledoni **(**Table 3).

Of the 402 sera analysed, 34.8% (140/402, 95% CI, 30.6–39.5) showed multiple reactions with different serogroups in the panel at a MAT titre ≥ 100, Table 4). Furthermore, following the acute case definition of leptospirosis in animals, which was confirmed by a fourfold increase in the MAT titre, probable acute leptospirosis with a MAT titre ≥ 800 was identified in 33.1% (133/402, 95% CI 28.6–37.8) of the animals.

**Table 3:** Seroprevalence of *Leptospira* serovars/serogroups by microscopic agglutination test (titre ≥ 100) among slaughter cattle (*N* = 402) sampled in the Bahr El Ghazal Region, South Sudan, from January to February 2023

Leptospira Serovar	Serogroup	MAT titre										95% CI	
		100	200	400	800	1600	3200	6400	12,800	Npos*serovar	Prevalence*	Lower	Upper
*L. borgpetersenii* sv Tarassovi	Tarassovi	35	84	72	70	34	18	3	0	316	78.6%	74.4	82.3
*L. borgpetersenii* sv Kenya	Ballum	14	38	17	12	1	0	0	0	82	20.4%	16.7	24.4
*L. kirschneri* sv Butembo	Autumnalis	17	11	4	2	0	1	0	0	35	8.7%	5.7	11.7
*L. interrogans* sv Pomona	Pomona	8	7	8	2	2	0	1	0	28	7.0%	4.5	9.5
*L. interrogans* sv Hebdomadis	Hebdomadis	9	7	3	1	0	0	0	0	20	5.0%	2.8	7.2
*L. borgpetersenii sv* Sejroe	Sejroe	7	2	0	0	0	0	0	0	9	2.2%	1.0	4.0
*L. kirschneri sv* Grippotyphosa	Grippotyphosa	1	2	3	2	0	0	0	0	8	2.0%	0.7	3.2
*L. borgpetersenii* sv Nigeria	Pyrogenes	2	1	2	1	1	0	0	0	7	1.7%	0.7	3.0
*L. interrogans* sv Australis	Australis	1	3	0	0	0	0	0	0	4	1.0%	0.0	2.2
*L. interrogans* sv Canicola	Canicola	1	0	0	0	0	0	0	0	1	0.2%	0.0	1.0
*L. interrogans* sv Icterohaemorrhagiae	Icterohaemorrhagiae	0	0	0	0	0	0	0	0	0	0.0%	0.0	1.0
*L. weilli* sv Celledoni	Celledoni	0	0	0	0	0	0	0	0	0	0.0%	0.0	1.0
		95	155	109	90	38	19	4	0	510			
Any *Leptospira* spp., serovar	Any positive excluding cross-reaction									329	81.8%	77.9	85.3

*This is an apparent prevalence since the microscopic agglutination test (MAT) is not 100% sensitive or specific

**Table 4:** *Leptospira* spp. serogroups that were involved in multiple exposures among the seropositive cattle sampled during a cross-sectional study in South Sudan

	Sej	Icter	Pom	Aut	Grip	Heb	Ball	Pyr	Cell	Aust	Tar	Can
Sej	Null	0	0	1	0	5	2	0	0	0	8	0
Icter	0	Null	0	0	0	0	0	0	0	0	0	0
Pom	0	0	Null	8	1	1	8	0	0	0	27	0
But	0	0	0	Null	3	3	5	2	0	0	29	0
Grip	0	0	0	0	Null	0	2	2	0	0	6	0
Heb	0	0	0	0	0	Null	8	1	0	1	17	0
Ball	0	0	0	0	0	0	Null	2	0	1	81	0
Pyr	0	0	0	0	0	0	0	Null	0	0	6	0
Cell	0	0	0	0	0	0	0	0	Null	0	0	0
Aust	0	0	0	0	0	0	0	0	0	Null	2	0
Tar	0	0	0	0	0	0	0	0	0	0	Null	1
Can	0	0	0	0	0	0	0	0	0	0	0	Null
Exposure to over two serogroups	Sej - Heb - Tar (3), Sej - Ball - Tar (1), Pom - Aut - Tar (4), Aut - Gri - Tar (1), Aut - Heb - Tar (1), Aut - Ball - Tar (2), Aut - Pyr - Tar (1), Gri - Ball - Tar (1), Heb - Ball - Tar (4), Ball - Pyr - Tar (1), Sej - Aut - Heb - Tar (1), Sej - Heb - Ball - Tar (1), Pom - Aut - Ball - Tar (1), But - Gri - Pyr - Tar (1), Pom - Aut - Gri - Ball - tar (1), Pom - Aut - Heb - Ball - Tar (1)											

Sej-Sejroe, Icter-Incterohaemorrhagiae, Pom-Pomona, Aut-Autumnalis, Grip-Grippotyphosa, Heb-Hebdomadis, Ball-Ballum, Pyr-Pyrogenes, Cell-Celledoni, Aus-Australis, Tar-Tarassovi, Can-Canicola

### Performance of the MAT against the lipL32 quantitative real-time polymerase chain reaction (PCR) assay

Out of the 400 urine samples examined using *lipL32* PCR, 23 samples were positive. This indicated an apparent prevalence of 6% (23/400, 95% CI 3.8–8.4). The epithelial calculator [40] was utilized with a 93% sensitivity and 98.3% specificity for lipL32 PCR [41] to estimate the true prevalence of 4.4% (95% CI 2.4–7.4). Among the 23 PCR-positive samples, 19 corresponding sera also tested positive with MAT. The 19 PCR positive samples were corresponding to MAT seropositivity against Pomona [1], Automnalis [3], Grippotyphosa [1], Hebdomadis [2], Ballum [3], Pyrogenes [1], and Tarassovi [18]. However, the overall agreement between the MAT and PCR results was observed to be very poor, as indicated by a Cohen’s kappa statistic of 0.001 (P-value = 0.913) (Table 5).

**Table 5:** Comparison of MAT and *lipL32* PCR results across (*N* = 400) serum and urine samples of slaughtered cattle in the Bahr El Ghazal Region, South Sudan, from January to February 2023

Serology	Molecular		Total
	lipL32 PCR - Positive	lipL32 PCR -Negative	
MAT - Positive	19	308	327
MAT - Negative	4	69	73
Total	23	377	400
Cohen’s kappa statistic (95% CI)	0.001(-0.020–0.022)		
P – value	0.913		

However, we found a moderate correlation between high MAT titres ≥ 800 and PCR positive results with Cohen’s kappa statistic of 0.501 (P-value < 0.001) (Table 6). MAT high titres ≥ 800 were observed against sv. (Tarassovi) sg. Tarassovi at titres 800 [4], 1600 [1], 3200 [2], 6400 [1].

**Table 6:** Correlation between MAT high titres ≥ 800 and PCR positivity across (*N* = 400) serum and urine samples of slaughtered cattle in the Bahr El Ghazal Region, South Sudan, from January to February 2023

Serology	Molecular		Total
	lipL32 PCR - Positive	lipL32 PCR -Negative	
MAT – Positive with titres < 800	15	377	392
MAT – positive with titres ≥ 800	8	0	8
Total	23	377	400
Cohen’s kappa statistic (95%CI)	0.501(0.287–0.714)		
P – value	0.001		

### Risk factors for seroprevalence of pathogenic Leptospira species

The risk factors associated with *Leptospira* spp. seroprevalence, including age, sex, breed, and BCS (body condition score), were investigated using bivariable logistic regression (Table 7**)**. Exposure variables with a P-value of less than or equal to 0.2 were considered for inclusion in the multivariable logistic regression analysis. These variables were retained in the model if the likelihood ratio test demonstrated statistical significance (P – value ≤ 0.05).

Moreover, a multivariable logistic regression analysis was conducted using the manual backwards Wald method. This approach aimed to evaluate the association between these exposure variables and *Leptospira* spp. seropositivity while controlling for the effect of other variables, hence predicting the final model.

**Table 7:** Population characteristics, seroprevalence of *Leptospira* spp. and associated risk factors analysed using bivariable and multivariable logistic regression models of sampled slaughtered cattle (*N* = 402) in the Bahr El Ghazal Region, South Sudan, from January to February 2023

				Bivariable							Multivariable					
Variables	Categories	*n* (%)	(Number positive) Prevalence%	OR	*P* value	95% C.I.				OR		*P* value		95% C.I.		
						Lower	Upper						Lower		Upper	
Age	Young (< 2 years)	127(31.6)	97(76.4)	Ref.					Ref.							
	Adult (≥ 2 years)	275(68.4)	232(84.4)	2.0	0.016	1.1	3.5		2.0			0.016	1.1		3.5	
Sex	Male	218(54.2)	168(77.1)	Ref.					Ref.							
	Female	184(45.8)	161(87.5)	2.1	0.008	1.2	3.5		2.1			0.008	1.2		3.6	
Breed	Nilotic	260(64.7)	205(78.8)	Ref.					Ref.							
	Felata/Mbororo	142(35.3)	124(87.3)	1.8	0.037	1.0	3.2		2.4			0.005	1.3		4.5	
BCS	Poor	83(20.6)	73(88.0)	Ref	0.192											
	Good	296(73.6)	236(79.7)	0.5		0.2	1.1									
	Very good	23(5.7)	20(87.0)	0.9		0.2	3.6									

Among the associated risk factors assessed for leptospires in slaughtered cattle via the multivariable logistic regression model (Table 7), age, sex, and breed had statistical significance (P -value ≤ 0.05) when controlling for the effect of other variables in the model.

Older animals (≥ 2 years) had 2.0 times greater odds (95% CI 1.1–3.5) of being seropositive than younger animals (< 2 years), P-value = 0.016. Compared with male animals, female animals had 2.1 times greater odds (95% CI 1.2–3.6) of being seropositive (P- value = 0.008), and Felata/Mbororo cattle had 2.4 times greater odds (95% CI 1.3–4.5) of being seropositive than did the Nilotic local breed (P -value = 0.005).

## Discussion

To our knowledge, this was the first cross-sectional study conducted to investigate the molecular and serological prevalence of *Leptospira* spp. in the Bahr El Ghazal Region of South Sudan. The significantly high seroprevalence of 81.8% coupled with a shedding prevalence of 6% of pathogenic leptospiral DNA in slaughtered cattle urine, suggest the potential involvement of cattle in transmitting *Leptospira* spp. within the region. These findings demonstrate the likelihood of leptospirosis being endemic in the study area and South Sudan as a whole.

Our study’s findings supported the hypothesis that *Leptospira* spp. pose a potential risk of exposure among cattle in the Bahr El Ghazal region and South Sudan. An earlier serological survey was conducted in the Upper Nile Province of South Sudan, and the reported seroprevalence of *Leptospira* spp. in cattle was 63.5% [14]. A comparatively lower seroprevalence of antibodies against *Leptospira* spp. has been documented elsewhere in East Africa. For instance, in Tanzania, 30.3% and 30.37% of the population were reported [29, 42]; in Uganda, 27.8% were reported [26]; and in Kenya, 25–34% were reported [37]. While these countries seem to have similar prevalences, the prevalence in South Sudan is much higher. The difference in prevalence may occur due to differences in climate, rainfall patterns, geography, animal husbandry and agricultural practices, presence of other maintenance hosts, herd density and inter- and intraspecies animal contact patterns and other unknown risk factors. Differences may also stem from study design variations, such as the chosen study population, sample size, serogroups/serovars included in the panels of diagnosis, and methodologies employed for screening. Moreover, knowledge about the disease show variations in prevalence.

It is crucial to interpret slaughterhouse-based results with caution due to inevitable biases. Antibody levels against *Leptospira* spp. tend to be greater in older animals than in young animals, and older animals are more prevalent in slaughterhouses across Africa. This trend has been reported among slaughtered cattle in Uganda [26]. Therefore, the seroprevalence observed in our study might have overestimated the prevalence within the general cattle population. We recorded a significantly high prevalence of sv. (Tarassovi) sg. Tarassovi at 78.6%, aligning with the findings of an earlier study in South Sudan’s Upper Nile Province, which reported a seropositivity of 50.6% against Tarassovi in cattle [14]. This finding strengthens the suggestion that Tarassovi remains the dominant serovar in South Sudan and that cattle are regularly exposed to this serovar. This cross-sectional study focused on a healthy population, the broader health, and economic impacts within cattle herds across South Sudan remain unknown. Notably, antibodies against Tarassovi were also prevalent in the majority of cattle in Uganda (11.6%) [26]. Moreover, Tarassovi’s adaptation to cattle as a maintenance host was highlighted in a systematic review of leptospirosis epidemiology in Africa [43]. Similar findings were also reported from neighboring Uganda [26, 27].

Among the other commonly reactive serovars, Kenya had a prevalence of 20.4%, followed by Pomona at 7.0%, Hebdomadis at 5.0%, Sejroe at 2.2%. Grippotyphosa at 2.0%, Nigeria at 1.7%, Australis at 1.0%, and Canicola at 0.2%. The relatively low prevalence of reactions to serogroup Sejroe in South Sudan and Kenya indicates that the serogroup distribution may vary geographically and local screening programs before starting a vaccination campaign are crucial, as serovar Hardjo of the same serogroup is very common in cattle in other areas of the world and an important component in cattle vaccines [44–46]. The observed similarities and variations in serovars might be attributed to shared environmental factors and their adaptation to natural animal hosts. This notion is supported by evidence of cross-border livestock trading between South Sudan, Uganda, Kenya, and other neighbouring countries [47, 48].

The sv and sg Icterohaemorrhagiae was seronegative in all the serum samples, which was similar to the findings reported in Uganda by Dreyfus et al. [27] and the low seroprevalence reported by Alinaitwe et al. [26], Additionally, the same serovar and serogroup were absent in rodents sampled from the slum urban settlements in Nairobi, Kenya [49]. The seronegative of this rodent-associated sv and sg Icterohaemorrhagiae could be explained by the fact that rodents may play a lesser role in contaminating the farmyards of rural areas where those animals are coming from, hence reducing the risk of exposure in animals; alternatively, rodents may be less infected or infected with other serovars in Africa. Similarly, the role of rodents in the contamination and transmission of *Leptospira* spp. to humans in urban slum residential areas has been well-studied [37]. To support the seronegative findings of sv and sg Icterohaemorrhagiae, we recommend rodent samples along with environmental ones to be considered in the future studies.

Antibodies against Celledoni were not detected in any of the serum samples; this negative result was in agreement with the findings of a systematic review of predominant *Leptospira* spp. in Africa, which reported no evidence of Celledoni [43], therefore, it could be left out in the MAT panel in future studies in cattle, when budget is an issue.

6% of the slaughtered cattle shed leptospires in their urine, based on our PCR test, with a comparably much greater seroprevalence of 81.8% detected from the same animals. Molecular detection of leptospiral DNA in urine is not always consistent with the serological findings, as the bacteria are excreted intermittently in urine for a few weeks to several months [50, 51], while antibodies can persist for months or years in the blood [52]. The excretion of leptospires in urine depends on the adaptability of serovars to different mammalian hosts known as maintenance hosts, which can be infected but without showing any clinical symptoms when acting as reservoirs [53, 54]. In addition, *Leptospira* spp. antibodies, as detected by MAT, persist in exposed animals for weeks to years while the level of leptospires in kidneys and consequently urine drastically reduces over time especially when treatment is administered [51].

Our findings are similar to the 5.8% urinary shedding status of slaughtered cattle reported from Uganda using PCR [18]. Utilizing a stochastic model and a binomial distribution, Alinaitwe et al. calculated that halal butchers and meat inspectors were at a daily 100% risk of being in contact with at least one shedding animal per day. Hence, these abattoir workers are exposed daily. If the abattoir workers in South Sudan slaughter a similar amount of cattle per day, they would have the same risk of exposure, given the similar prevalence in cattle [18]. Many abattoir studies proved a risk of infection with leptospires for slaughter workers, i.e. in Nigeria, [55], in Cameron [56] and as well in New Zealand [57]. Hence the slaughter workers in South Sudan are potentially at a high risk of contracting leptospirosis in the slaughterhouse.

A few studies have been conducted in East African countries using PCR to detect pathogenic leptospiral DNA from either urine or kidneys of cattle using *lip*L32. A *Leptospira* spp. prevalence of 7.1% was detected in the urine of slaughtered cattle in Tanzania [34] and of 1.8% in the urine of cattle in peri-urban areas of Addis Ababa in Ethiopia [58]. A relatively higher prevalence of *Leptospira* spp. DNA was reported from South Africa in kidney tissue samples of slaughtered cattle at 26.9% [59].

The main reason for the observed variations could be the seasonal effects on *Leptospira* spp. dynamics, as heavy rainfall and flooding increase the occurrence of leptospirosis, or there are more *Leptospira* spp. circulating [60]. Our sample collection was performed during the dry season, which might have contributed to the demonstrated lower prevalence.

Even though we found a poor agreement between MAT seropositivity and PCR positivity results, there was a moderate correlation between high MAT titres (≥ 800) and PCR positivity. The poor agreement between MAT and PCR for seropositivity is consistent with previous reports [61–63]. However, our findings of moderate agreement between higher MAT titres (≥ 800) and PCR align with this one study [64] but contrast with a report of consistently poor agreement between MAT and PCR even after adjusting for high MAT titres (≥ 800) [63]. Hence, there is no consistency in findings in the literature. This suggests that high MAT titres may have the potential to detect shedding animals and this finding should be further explored.

Taken together, the high seroprevalence of Tarassovi, the detection of antibodies against several serovars and the confirmed shedding of cattle in South Sudan demonstrated by detection of leptospiral DNA in urine, may imply a potential risk to humans to contract leptospirosis and highlights the public health importance of leptospirosis in South Sudan. The same hypothesis of a cattle–human transmission pathway was drawn by Dreyfus et al. in 2016, where a high prevalence of antibodies against sv. Nigeria at 19.8%, and the cattle-associated sg. Sejroe at 5.6% was reported among health centre patients in Hoima District, Western Uganda [27]. It is therefore recommended to investigate the role of leptospirosis in fever patients in regions with a high cattle prevalence in South Sudan, as Tarassovi was found to contribute to the large proportion of patients with unidentified fever in East Africa [65]. We also recommend conducting a study in abattoir workers to estimate the risk of infection and develop recommendations on possible protective measures, such as increasing the awareness and wearing personal protective equipment.

Among the risk factors identified in our study was old age, where older animals were found to be 2.0 times more likely to be seropositive than young animals. This could be because of continuous exposure throughout their long life and the persistence of antibodies in their blood [26, 66]. The sex of the animals was also found to be significantly associated with *Leptospira* spp. antibody seropositivity, with female cattle being 2.1 times more likely to be seropositive than male cattle. This could be because venereal transmission through semen is possible, with multiple females being infected by one male. A higher prevalence among females was also found in other studies [2, 56, 67].

Recent studies reporting a relatively high incidence of genital infections in animals from arid regions suggest that venereal transmission may serve as an alternative route for the spread of leptospirosis, particularly in environments where external conditions are unfavorable for the survival of leptospires outside the host. This alternative pathway warrants further investigation to fully understand its impact and prevalence in the epidemiology of the disease [68, 69].

Another risk factor found to be significantly associated with *Leptospira* spp. seropositivity was the breed of cattle, with the Mbororo breed of cattle being 2.4 times more likely to become seropositive than the local Nilotic breed.

The Mbororo cattle breed belongs to the nomadic pastoralists, and the herders migrate throughout West African countries from Libya, Mali, Niger, Chad, the Democratic Republic of Congo *(*DRC*)*, and Central Africa Republic *(*CAR*)* and to East African countries of Sudan and South Sudan during the dry season to trade (primarily cattle) in large urban centres [48], including Wau town of Western Bahr El Ghazal. This long journey may expose them to more diseases, including leptospirosis, as also reported by [20], compared to local breeds, as they contact wildlife and share the same pastures with other cattle herds, potentially contaminated rivers, and water streams.

This study was conducted in one slaughterhouse, as this was the largest within the region in terms of daily volume, and the animals brought to this slaughterhouse originated from all over the Bahr El Ghazal Region, which includes Mbororo cattle from West African countries. The findings of this study may not reflect the true status of leptospirosis at the herd level, as single animals from different herds might have been brought to the slaughterhouse. We could not determine the spatial distribution of leptospirosis due to the difficulty in tracing the origin of the animals because of widespread cattle raiding within the region and South Sudan at large.

## Conclusion

This study demonstrated that cattle slaughtered in the Bahr El Ghazal Region of South Sudan were exposed to pathogenic *Leptospira* spp. The high seroprevalence of *Leptospira* spp. suggests that leptospirosis may have significant socioeconomic implications for livestock-keeping communities, as it poses an unrecognized zoonotic health threat to slaughterhouse workers, farmers, the public and other livestock species. This was also confirmed by the detection of leptospiral DNA in the urine of slaughtered cattle. Based on the findings from this study, we recommend further studies on *Leptospira spp*., including those of febrile patients, abattoir workers and on-farm individuals, and this should involve collection of rodent and environmental samples to elucidate disease epidemiology and socioeconomic burden in animal hosts and humans in the region and South Sudan. This should be based on a One Health approach that calls for multidisciplinary, well-coordinated strategies geared toward the control and management of the disease in animals and humans to promote public health.

## Data Availability

No datasets were generated or analysed during the current study.

## Abbreviations

MAT: Microscopic Agglutination Test
OR: Odds Ratio
sv: Serovar
sg: serogroup
CI: Confidence Interval
CIDIMOH: Climate Change and Infectious Diseases Management - A One Health Approach
BCS: Body Condition Score
COVAB: College of Veterinary Medicine, Animal Resources and Biosecurity
UBG: University of Bahr El Ghazal
RT- qPCR: Real Time quantitative Polymerase Chain Reaction
DNA: Deoxyribo -Nucleic Acid
